# Obstructive Sleep Apnea-hypopnea Syndrome as a Novel Potential Risk for Aging

**DOI:** 10.14336/AD.2020.0723

**Published:** 2021-04-01

**Authors:** Yayong Li, Yina Wang

**Affiliations:** ^1^Department of Emergency, The Third Xiangya Hospital of Central South University, Changsha, China.; ^2^Department of Geriatrics, The Second Xiangya Hospital of Central South University, Changsha, China.

**Keywords:** obstructive sleep apnea-hypopnea syndrome, aging, pathophysiological mechanism, aging-associated diseases

## Abstract

Obstructive sleep apnea-hypopnea syndrome (OSAHS) is a common sleep disorder, negatively influencing individuals’ quality of life and socioeconomic burden. In recent years, OSAHS has been reported in not only constituting an aging-associated disease, but also in accelerating and/or potentiating aging mechanisms. However, the negative impacts of OSAHS on aging are underestimated because of low level of public awareness about this disease and high rates of undiagnosed cases, which are more critical in developing countries or economically disadvantaged regions. Hence, reviewing previously reported observations may assist scholars to better indicate that OSAHS is likely a novel potential risk for aging. Further understanding of the pathophysiological mechanism of OSAHS and its role in procession of aging may markedly highlight the importance of this common sleep disorder.

## Introduction

Aging is a complex, multifaceted process that induces a myriad of physiological changes over an extended period of time. Aging is accompanied by major biochemical and biomechanical changes at macroscopic and microscopic scales that affect not only tissues and organs, but also cells and subcellular organelles. To date, diseases are the main causes of death or shortening of lifespan in elderly, partly accounting for old-age mortality, including arteriosclerosis, diabetes, dementia, osteoporosis, osteoarthritis, and cancer.

Obstructive sleep apnea-hypopnea syndrome (OSAHS) is one of the most common sleep disorders, influencing middle-aged and old people with a reported prevalence of 5-14% [1]. Approximately 25% of men and 13% of women suffer from OSAHS worldwide [2]. This common sleep disorder is characterized by recurrent episodes of upper airway collapse, resulting in recurrent arousal from sleep and intermittent hypoxia (IH). IH and sleep fragmentation are the major pathophysiologic characteristics of OSAHS [3]. It is well known that untreated OSAHS may lead to several aging-associated diseases, such as coronary heart disease, hypertension, stroke, arrhythmia, diabetes, and metabolic syndrome [4-6]. IH is the main trigger of oxidative stress, systemic inflammation, and sympathetic activation, playing a role in the OSAHS-related mortality [7, 8]. Previous studies found that approximately 40% of untreated severe OSAHS patients died during a follow-up period of 8 years [9-11].

As a result, OSAHS may be a crucial disease, contributing to aging and aging-associated diseases. An effective early diagnosis and therapy of OSAHS may cause delay in progression of aging or certain aging-associated diseases through halting the progression of cellular and molecular changes. In recent decades, several studies have concentrated on the influences of OSAHS on hallmarks of aging in cellular and molecular levels to better understand the development of OSAHS-associated impairments and age-related comorbidities. This research has provided more knowledge regarding the influence of OSAHS on aging mechanisms, and further clarified how OSAHS may putatively accelerate aging and aging-associated diseases. We herein searched for relevant clinical studies published in the recent 20 years at PubMed and Web of Science databases with “OSAHS” and “aging” as keywords.

### OSAHS and aging-associated diseases

There are several common aging-associated diseases, including cardiovascular diseases, stroke, Alzheimer’s or Parkinson’s diseases, dyslipidemia, type 2 diabetes mellitus, obesity, metabolic syndrome, non-alcoholic fatty liver disease, and cancer [12-14]. In recent decades, untreated OSAHS has been widely reported to be linked with development of aging-associated diseases.

A previous research pointed out the correlation of untreated OSAHS with the aging-associated diseases [15]. Meanwhile, OSAHS was found as an independent risk factor for a verity of aging-associated diseases. The main pathogenic mechanism, causing systemic complications in OSAHS patients is believed to be IH-induced intermediary metabolism, and it depends on the burden of oxidative stress during sleep [16-18].

Hence, further research needs to be conducted to better understand the primary causes of aging and its relationship with aging-associated diseases (e.g., OSAHS).

### OSAHS and the main hallmarks of aging

Understanding the molecular mechanism of aging-related processes can extend the organism’s health span. The process of aging is characterized by progressive functional impairment and decreased capacity to respond appropriately to environmental stimuli and injuries. The aging processes cause dysfunction and impaired repair capacity of lung epithelial cells and fibroblasts [12, 19]. There are numerous mechanisms, playing a role in cellular aging, which were previously considered as the hallmarks of aging [20]. In recent years, a great number of studies demonstrated that OSAHS may contribute to the hallmarks of aging.

### OSAHS and genomic instability

A variety of genetic and environmental factors may influence aging, and a common denominator of aging is mitochondrial dysfunction and accumulation of genetic damage throughout life. IH is a major pathophysiologic feature of OSAHS patients that may result in cellular damage [21]. Studies found that the level of DNA damage is higher in OSAHS patients than that in healthy individuals. For instance, Kim et al. [22] reported that mitochondrial DNA copy number (mtDNA-CN) is lower in genomic DNA isolated from whole blood of OSAHS patients, which may be related to severity of OSAHS, reflecting excessive oxidative stress in OSAHS patients. Kontogianni et al. [23] compared basal DNA damage induced by hydrogen peroxide, ethanol, and gamma-irradiation in lymphocytes from OSAHS patients and healthy controls, and found that lymphocytes isolated from OSAHS patients had higher basal levels of DNA damage and were more sensitive to the effects of the DNA-damaging agents than lymphocytes collected from healthy controls. Xie et al. [24] pointed out that the mean frequency of binucleated cells with micronuclei was noticeably higher in OSAHS patients than that in healthy controls (P<0.01), and the frequency of micronuclei significantly differed among patients in mild, moderate, and severe stages (P<0.05). The mean frequency of nucleoplasmic bridge in OSAHS patients was also higher than that in healthy controls (P<0.05). Additionally, in patients with OSAHS, the mean frequency of binucleated cells with micronuclei was remarkably higher than that in healthy controls, and the frequency of micronuclei markedly differed among patients in mild, moderate, and severe stages. These results may be related to oxidative stress induced by IH and may be involved in the mechanisms of target organ damage caused by cardiovascular disease. IH can inhibit the proliferation of BV2 cells, as well as causing DNA damage, which may be one of the underlying mechanisms of hippocampal neuronal damage related to OSAHS [25].

Hypoxia-induced oxidative stress can lead to DNA damage, which is associated with chromosomal aberrations and micronuclei formation. The presence of oxidative stress seems to be the main cause of change in mtDNA-CN. In OSAHS patients, the presence of a persistent IH maintains excessive reactive oxygen species (ROS) in the cell, inducing cell injury, including mitochondrial dysfunction, mtDNA-CN alteration, and other negative influences [26].

### OSAHS and telomere attrition

Telomeres are long tracts of DNA at the linear chromosomes’ ends composed of tandem repeats of a guanine-rich sequence motif that vary in length from 2 to 20 kb, according to species [27]. In humans, the distribution of telomere length (TL) among different chromosome arms is heterogeneous. TL is reduced at a rate of 50-150 bp at each cell division in human somatic cells. Consequently, individual telomere shortening rate may be different in different cell lineages [28]. Thus, TL is a useful biological marker for aging process. When the cell cycle is arrested after telomeres reach a critical size, cells then become senescent or undergo apoptosis [29]. There is a growing body of literature suggesting that shorter TL is associated with over 30 different metabolic and inflammatory diseases [3]. Several studies reported the association of OSAHS with short TL, and demonstrated that shortening TL is more predominant in patients with moderate and severe OSAHS [3, 30-33].

As a community-based cohort study, the Korean Genome and Epidemiology Study (KoGES) consortium demonstrated a significant association between leukocyte telomere length (LTL) and elevated narrow-band low frequency coupling (e-LFC_NB_) via interacting with OSAHS severity (P <0.0001) [34]. Carroll et al. [35] assessed whether sleep apnea is related to LTL in the Multi-Ethnic Study of Atherosclerosis (MESA), and found that severe OSAHS (AHI > 30) was cross-sectionally associated with shorter LTL (P = 0.007) when adjusting for ethnicity/race, age, sex, body mass index (BMI), smoking, and physical activity, while these effects were diminished after treating OSAHS. Riestra et al. [3] reported that LTL varied by OSAHS risk in African-American women (0.532 ± 0.006 vs. 0.569 ± 0.008, P = 0.04), and confirmed that women who were at higher risk of OSAHS presented shorter LTL compared with those who were at lower risk, independent of age, hypertension, obesity, smoking, alcohol consumption, level of education, and income. A meta-analysis that included 8 eligible studies and 2639 participants showed that shortened TL was noticeably associated with OSAHS (95% confidence interval (CI): -0.06-0.00; P = 0.003 with I-square of 85%) [36].

Therefore, risk of OSAHS may contribute to the acceleration of aging process through shortening TL. Studies discussed the mechanisms of shortened TL in OSAHS. Tempaku et al. [30] demonstrated that hypoxia is the most important OSAHS-related factor associated with shortened TL, and found that OSAHS may result in attrition of TL and promote acceleration of aging. IH due to OSAHS is likely a major contributor to shortened TL in middle-aged men [34-37]. Kim et al. [32] pointed out that the serum concentration of hydrogen peroxide was markedly higher in OSAHS patients, which was closely associated with the severity of OSAHS. The shortened TL in OSAHS patients was found to be inversely correlated with the concentration of hydrogen peroxide and was also associated with severity of OSAHS. The above-mentioned results provided an evidence that shortened TL or excessive cellular aging may be distinctive in circulating leukocyte of OSAHS patients and may be a predictive biomarker for reflecting the burden of oxidative stress in the peripheral blood of OSAHS patients. Oxidative stress was presented as a significant modulator of telomere maintenance, and telomere-driven replicative senescence is a stress response [30, 38].

## OSAHS and epigenetic alterations

Epigenetics refers to the study of heritable phenotype changes that do not involve alterations in the DNA sequence, including three related molecular mechanisms for genome regulation: DNA methylation, non-coding RNAs (ncRNAs), and histone modifications. Epigenetic processes regulate early cellular differentiation through making interactions between genes and the environment, modulating gene transcription, leading to changes in cellular phenotype [39, 40]. OSAHS is characterized by phenotypic variations, which can be partly attributed to specific gene polymorphisms. Recently, the role of epigenetics in the pathogenesis of OSAHS has noticeably attracted scholars’ attention in order to provide a more precise perception about clinical phenotyping and targeted therapies.

### OSAHS and DNA modification

DNA methylation is a well-characterized epigenetic biochemical process that typically occurs by the covalent addition of a methyl group at the 5-carbon of the cytosine ring, resulting in 5-methylcytosine, changing the appearance and structure of DNA, without altering the primary DNA sequence [41]. The most studied DNA modification occurs at the 5th position of the pyrimidine ring in the sequence context of cytosine followed by guanine (CpG) [42, 43].

Further evidence demonstrated that epigenetic changes, defined as heritable phenotype changes that did not involve alterations in the DNA sequence, were found to be associated with the pathogenesis and development of OSAHS. Kheirandish-Gozal et al. [44] studied 36 OSAHS children (OSAHS group) and 35 children in control group, and observed a CpG site located at the proximal promoter region of endothelial nitric oxide synthase (eNOS) gene, approximating important transcriptional elements, and reported a significantly higher methylation level in OSAHS group compared with that in control group. The above-mentioned findings indicated that hypermethylation of the core promoter region of eNOS gene in OSAHS children could be related to decreased eNOS expression. Kim et al. [45] suggested that the numerical impairment of regulatory T cell (Tregs) in pediatric OSAHS patients could be attributed to hypermethylation of FOXP3, thereby inducing an imbalance of Th1/Th2 cytokines. Khalyfa et al. [46] found that IH during late pregnancy may increase the propensity for metabolic dysregulation and obesity in adult offspring via epigenetic modifications, providing a comprehensive illustration of the adverse epigenetic and metabolic consequences associated with OSAHS in offspring during pregnancy. More than 90% of patients with obesity hypoventilation syndrome (OHS) have concomitant OSAHS. Cortese et al. [47] compared DNA methylation before starting PAP treatment (PRE group) and after PAP treatment (POST group) in 15 adult OHS patients. Among 1,847 regions, significant differential DNA methylation was noted between the two groups (P < 0.001; model-based analysis of tiling arrays score > 4), and DNA methylation was particularly increased at the PPAR-responsive elements (PPAREs) of 8 genes in the post-treatment samples (PRE/POST fold changes: ADIPOQ, 1.73; PEPCK, 9.27; NOS2, 7.78; HMOX, 2.74; FABP4, 2.49; CD36, 5.04; ABCG1, 1.72 and ABCA1, 3.11). This suggested that PAP treatment leads to an increase in DNA methylation at PPAREs, and epigenetic regulation of PPAR pathways in macrophages may operate as a major component of OSAHS, leading to macrophage activation.

### OSAHS and non-coding RNAs

Another epigenetic regulation is given by non-coding RNAs (ncRNAs). The most studied ncRNAs include some types of RNA molecules which may have an influence on genome regulation, which are not coded for proteins, such as long non-coding RNAs (lncRNA), microRNAs (miRNA), and small interfering RNAs (siRNA). LncRNAs are defined as RNA transcripts with length exceeding 200 nucleotides that are not translated into proteins. A miRNA is a small non-coding RNA molecule (containing about 22 nucleotides) found in plants, animals and some viruses, playing significant roles in RNA silencing and post-transcriptional regulation of gene expression [48, 49].

Results of recently conducted studies indicated that the presence of hypertension, atherosclerosis, and endothelial dysfunction in OSAHS may be associated with upregulation of miR-574 and miR-130a expressions, while it downregulates the expressions of miR-630, miR-199, miR-107, miR-664a, and miR-485 [50-54].

Studies found that several miRNAs could influence intermittent hypoxia with re-oxygenation process and affect hypoxia-induced cell apoptosis in end organs. Several miRNAs up-regulated by hypoxia are direct targets of hypoxia-inducible factor-1β (HIF-1β), HIF-2β, nuclear factor kappa B (NF-κB), or their responsive genes, and possess a positive feedback loop to stabilize HIF-1β protein, while other miRNAs downregulated by hypoxia commonly suppress the expression of HIF or inflammatory signaling, and can be engaged in protective mechanisms against IHR injury. Upregulation of miR-155, miR-31, miR-21, miR-26b, and miR-218, and downregulation of miR-452, miR-203, miR-145, miR-223, miR-207, and miR-365 may contribute to the development of chronic IH (CIH)-induced cardiovascular remodeling, insulin resistance, cognitive dysfunction, and chronic kidney failure [55].

### OSAHS and histone modifications

As the third epigenetic mechanism, histone modifications include methylation, acetylation, phosphorylation, ubiquitination, sumoylation, citrullination, and ADP-ribosylation. Histone modifications contribute in a larger dynamic process, regulating accessibility of DNA and transcription [15, 56]. The nucleosome core is composed of two H2A-H2B dimers and a H3-H4 tetramer, with the latter protruding long tails that can be covalently modified with acetylation or methylation. Generally speaking, highly acetylated histones tend to associate with active transcription. Deacetylated histones, in contrast, contribute to gene silencing. Histone methylation can be associated with either transcriptional repression or activation, depending on the position of residues modified and the number of methyl groups [2, 57, 58].

Previous studies indicated a pivotal role of H3K4me3 and H3K9me3 over the promoter regions in epigenetic activation of the NOX-1, -2, -4, and -5 genes for cellular senescence and endothelial dysfunction [59, 60]. Recently, it was suggested that sustained hypoxia can upregulate the oxygen-dependent demethylases (KDMs), which may increase demethylation of methylated lysine residues, including H3K9me3 and H3K4me3 [2, 61-63]. Another study showed histone modifications in oxidative stress pathways in aorta macrophages in mice exposed to IH [64]. Macrophages isolated from aortas of mice exposed to CIH showed over-representation of the active histone mark (H3K9ac) in pro-inflammatory and oxidative stress signaling pathways, such as HIF-1, p53, NF-kB, transforming growth factor-β (TGF-β), Forkhead box protein O4 (FOXO4), and IL-6, while genes associated with over-representation of the repressive histone mark (H3K27me3) were found relevant to anti-inflammatory and glutathione redox pathways. The above-mentioned outcomes demonstrated that histone modification may be involved in the establishment of the IH-induced endothelial dysfunction and cellular senescence in OSAHS [2].

### An association among miRNA, DNA methylation, and histone modification under hypoxic stimulus

Emerging evidence suggested that DNA methylation is an epigenetic mechanism of gene silencing achieved through the addition of methyl groups to cytosines within CpG dinucleotides, frequently present in clusters in the genome. A number of hypoxia-related miRNAs can be regulated by aberrant DNA methylation. DNA methylation of the CpG islands in the promoter region of miR-124-3p was enhanced under hypoxic conditions [65]. Hypoxia-induced miR-210 promoter demethylation enhances proliferation, autophagy, and angiogenesis of Schwannoma cells. An association among miRNA, DNA methylation, and histone modification may suggest potential biomarkers and targeted therapies for OSAHS [66].

## OSAHS and loss of proteostasis

Proteostasis is achieved by the coordinated action of several proteins known collectively as the proteostasis network. Under normal conditions, the proteostasis networks rapidly sense and rectify disturbances in the proteome to restore basal homeostasis [67]. The ability of several cells and organs to preserve proteostasis under resting and stress conditions is gradually compromised with age [68]. Numerous lines of evidence supported a tight relationship between proteostasis and healthy aging, and loss of proteostasis is partly associated with the pathogenesis of diverse aging-associated diseases.

Such networks include lysosomes, the autophagy machinery, molecular chaperones, translational machinery, and ubiquitin-proteasome system [69]. The lysosome-autophagy and the ubiquitin-proteasome systems may play a role in proteostasis maintenance. However, further studies need to be carried out to indicate whether these components may refold into their original stable conformation or may be eliminated from the cell through proteolysis [70].

OSAHS may increase oxidative stress, which may alter proteostasis networks and contribute to aging and aging-associated diseases. Only a limited number of studies have explored the role of proteostasis in the OSAHS-related aging, which have mainly concentrated on molecular heat shock proteins (HSPs). They are a large family of evolutionarily conserved molecular chaperones with pivotal roles in cell survival and development. HSPs can be broadly classified into two families based on comparable molecular mass.

Studies reported relationships between hypertension and HSP72 in OSAHS patients. Additionally, the relatively lower HSP72 level in the morning may be related to the prognosis of cerebrovascular events in OSAHS patients [71]. This could indicate a protective mechanism of HSP72 against oxidative stress derived from OSAHS. HSP70 was found to be upregulated in patients with OSAHS [72]. However, severe OSAHS patients expressed significantly lower level of HSP70 in peripheral blood mononuclear cells (PBMCs) than controls, which was no longer observed upon continuous positive airway pressure (CPAP) therapy. These findings suggested that repetitive stress during sleep in untreated patients may lead to HSP70 exhaustion, which may induce structural changes and protein modifications [72-75].

IH may be a reason for reduction of proteasomal activity in cells obtained from the hippocampus in animal studies. Similarly, CIH induced endoplasmic reticulum (ER) stress and unfolded protein response in the hippocampus and prefrontal cortex [76]. Moreover, sleep fragile (SF) may play a substantial role in OSAHS-mediated proteostasis impairments. Misfolded proteins can accumulate within ER, causing ER stress with subsequent activation of the unfolded protein response. In the hippocampus of mice, SF was also reported to blunt the circadian rhythm of autophagy-related proteins [77].

Alzheimer’s disease (AD) is characterized clinically by a progressive and gradual decline in cognitive function and neuropathologically by the presence of neuropil threads, specific neuron loss, and synapse loss in addition to the hallmark findings of neurofibriallary tangles and senile plaques. OSAHS may lead to early changes in the biomarkers of AD, including a wide array of cerebrospinal fluid and blood biomarkers, such as Aβ, tau proteins, and acute-phase proteins [78, 79].

Several studies assessed a possible role of OSAHS in metabolic disorders, such as glucose intolerance and type 2 diabetes mellitus [80]. Compared with healthy controls, mild-to-severe OSAHS patients showed insulin resistance and glucose intolerance. Insulin sensitivity of moderate-to-severe OSAHS patients with CPAP treatment was reported to rapidly improve compared with non-treated patients [81, 82].

Compared with healthy controls or mild OSAHS patients, patients with moderate-to-severe OSAHS have also demonstrated impairments in pancreatic β-cell function. As the main OSAHS-associated mechanism, IH may trigger these impairments [83].

## OSAHS and mitochondrial dysfunction

The mitochondria, which are main sources for the formation of ROS from electron transport chain (ETC), are involved in several fundamental cellular processes [84]. The mitochondria are susceptible to hypoxia, because under normoxic conditions, mitochondrial respiration consumes greater than 90% of the oxygen in humans. In response to sustained hypoxia, mitochondria consume almost all the oxygen and remove free cytosolic oxygen, causing rapid stabilization of HIF-1α. Hypoxia-inducible factor-1 (HIF-1) activates genes, playing a significant role in vascular function, including vascular endothelial growth factor (VEGF), erythropoietin (EPO), and inducible nitric oxide synthase (iNOS) [85, 86]. These factors increase tissue perfusion and oxygenation, and participate in the adaptive response to hypoxia, facilitating recovery from the initial hypoxic insults. Mitochondrial dysfunction can influence a wide range of important cellular functions, and can lead to a variety of aging-associated diseases [26].

Kim et al. [22] compared mtDNA-CN between 20 healthy volunteers and 20 OSAHS patients, and observed that mtDNA-CN is lower in whole blood DNA of OSAHS patients, which may be related to OSAHS severity, reflecting excessive oxidative stress in patients with OSAHS. IH in OSAHS patients may lead to dysfunction of mitochondria and endoplasmic reticulum and activation of nicotinamide adenine dinucleotide phosphate oxidase (NADPH oxidase), which may eventually cause overproduction of ROS [84]. Additionally, IH possibly results in activation of NF-κB through mitochondrial stress, triggering the production of tumor necrosis factor-α (TNF-α) or other inflammatory mediators. IH may cause a delayed increase in HIF-1α, leading to the activation of NFκB-driven inflammation, possibly as a result of oxidative stress [86-89].

The process of mitochondrial dysfunction in OSAHS patients may be able to make mitochondria become a representative biomarker for adverse outcomes from oxidative stress and some aging-associated diseases. The presence of mitochondrial dysfunction in OSAHS patients could be one of the mechanisms, contributing to intensify other aging-related diseases frequently associated with OSAHS, involving cardiovascular, metabolic, and inflammatory diseases [26].

## OSAHS and stem cell exhaustion

Decline in stem cell function causes loss of tissue homeostasis and increased incidence of aging-associated diseases. Besides, combination of long-term exposure to aberrant niche signals, incorrect activation of developmental pathways, and loss of stem cell identity may underlie the loss of regenerative capacity in aging [90].

Oxidative stress and inflammation can mobilize adult stem cells from the bone marrow into the circulating blood, and adult stem cells can differentiate into specialized cell types of an organ or tissue. The limited available data evaluated three stem cell niches within the OSAHS context: endothelial progenitor cells (EPCs), mesenchymal stem cells (MSCs), and very small embryonic-like stem cells (VSELs)[91]. Jelic et al. [92] found that level of an apoptosis marker in endothelial cells was increased in mild-to-severe OSAHS patients compared with healthy subjects. OSAHS alone impairs endothelial repair capacity and promotes endothelial apoptosis, which may clarify accelerated atherosclerosis and increased cardiovascular risk in OSAHS patients. Carreras et al. [93] studied 30 rats with OSAHS and 30 healthy rats. Compared with control serum, apneic serum showed a significant increase in chemotaxis in MSCs, and adhesion of MSCs to endothelial cells was greater. Compared with serum from healthy rats under normoxia, both MSC adherence and endothelial repair capacity on monolayers of cultured endothelial cells from rat aorta increased [91]. Gharib et al. [94] assessed the potential role of VSELs in OSAHS using an IH-based animal model. Compared with normoxic controls, a six-fold increase was noted in the blood/bone marrow ratio of VSELs in those animals exposed to IH, which may be evidence of mobilized VSELs from bone marrow into peripheral blood. Further studies demonstrated the increased plasma levels of some factors, such as hepatocyte growth factor (HGF), stromal cell-derived factor-1 (SDF-1α), and leukemia inhibitory factor (LIF), which could partly explain the recruitment of VSELs from bone marrow to peripheral blood in response to IH [94, 95].

## OSAHS and inflammation involved in altered intercellular communication

Beyond cell-autonomous alterations, aging also involves changes at the level of intercellular communication. Inflammation is one of these prominent aging-associated alterations in intercellular communication. Inflammation may result from multiple causes, including propensity of senescent cells to secrete proinflammatory cytokines, failure of an ever more dysfunctional immune system to effectively clear pathogens, and dysfunctional host cells.

Inflammatory responses play a substantial role in the pathogenesis of numerous aging-associated diseases (e.g., coronary heart disease, obesity, and type 2 diabetes) [96], and the majority of these diseases are complications of OSAHS.

A great number of previous researches reported increased plasma and serum levels of several systemic inflammatory markers in patients with mild, moderate and severe OSAHS compared with healthy controls, including TNF-α, C-reactive protein (CRP), IL-8, IL-6, intercellular adhesion molecule-1 (ICAM-1), and vascular cell adhesion molecule-1 (VCAM-1) [97]. In particular, NF-κB expression was elevated in patients with OSAHS [98]. NF-κB is a key transcription factor, playing a role in inflammatory cascades, and overactivation of the NF-κB pathway is one of the transcriptional signatures of aging [20]. Inflammatory and stress responses were found to be associated with activating NF-κB in the hypothalamus, as well as inducing a signaling pathway that results in reduced production of gonadotropin-releasing hormone (GnRH) by neurons. The reduction of GnRH can contribute to numerous aging-related changes, such as muscle weakness, skin atrophy, bone fragility, and reduced neurogenesis, which indicated a novel association between inflammation and aging [99]. Sirtuin 1 (SIRT1) is a protein involved in some aging processes, including DNA damage repair, cell cycle regulation, and telomere attrition, playing a role in downregulation of inflammation-related genes. Compared with healthy controls, the level and activity of SIRT1 were found to be reduced in PBMCs from moderate-to-severe OSAHS patients, and a lower SIRT1 level may also contribute to the potential effect of OSAHS on aging [100-103]. Li et al. reported that the expressions of genes associated with AD, such as CCL2, IL6, CXCL8, HLA-A, and IL1RN in patients with severe OSAHS were significantly different from those in patients with non-severe OSAHS [104]. Elevated serum levels of IL-6, IL-1ß, and MCP-1 in patients with OSAHS may be risk factors for the development of AD as well [105-107].

## Conclusions

Aging-associated accumulation of cellular damages and degenerative changes may lead to dysfunction and failure at the tissue levels, ultimately resulting in aging-associated diseases. In recent decades, OSAHS has been observed in not only constituting an aging-associated disease, but also in accelerating and/or potentiating aging mechanisms. Due to its crucial role in the process of aging and aging-associated diseases, OSAHS has significantly attracted scholars’ attention. During recent decades, several studies have provided relevant insights into OSAHS potential contribution to the development of aging and aging-associated diseases. These findings involve nine cellular and molecular hallmarks, in which telomere attrition plays a substantial role in OSAHS-associated aging, which was supported by several large-scale clinical studies. Review of these observations could assist us to conclude that OSAHS is likely a novel potential risk for aging.

OSAHS is a common health problem, negatively affecting individuals’ quality of life and socioeconomic burden. Early effective diagnosis and therapy of OSAHS may prevent IH-induced progression of cellular and molecular changes that may hopefully cause delay in progression of aging and certain aging-associated diseases. However, the negative influence of OSAHS on aging is underestimated because of the low level of public awareness about this disease and high rates of undiagnosed cases, which are more critical in developing countries or economically backward regions. Further understanding the pathophysiological mechanism of OSAHS and its role in aging procession may noticeably highlight the importance of this common sleep disorder.
